# Translational research in pediatric urology: methods of investigation of urogenital system in human fetuses

**DOI:** 10.1590/S1677-5538.IBJU.2023.9903

**Published:** 2023-05-29

**Authors:** Luciano A. Favorito, Francisco José Barcellos Sampaio

**Affiliations:** 1 Unidade de Pesquisa Urogenital Universidade do Estado do Rio de Janeiro Rio de Janeiro RJ Brasil Unidade de Pesquisa Urogenital - Universidade do Estado do Rio de Janeiro - Uerj, Rio de Janeiro, RJ, Brasil,; 2 Serviço de Urologia Hospital Federal da Lagoa Rio de Janeiro RJ Brasil Serviço de Urologia, Hospital Federal da Lagoa, Rio de Janeiro, RJ, Brasil

## COMMENT

Knowledge of the structure of the urogenital organs in human fetuses is of great importance for understanding the main congenital anomalies. In this editorial, we will comment on the main study methods carried out in basic research on human fetuses in our unit, with the aim of bringing information that will help in the diagnosis and treatment of anomalies of the kidney, ureter, bladder, urethra, penis and testicle.

**Figure 1:**
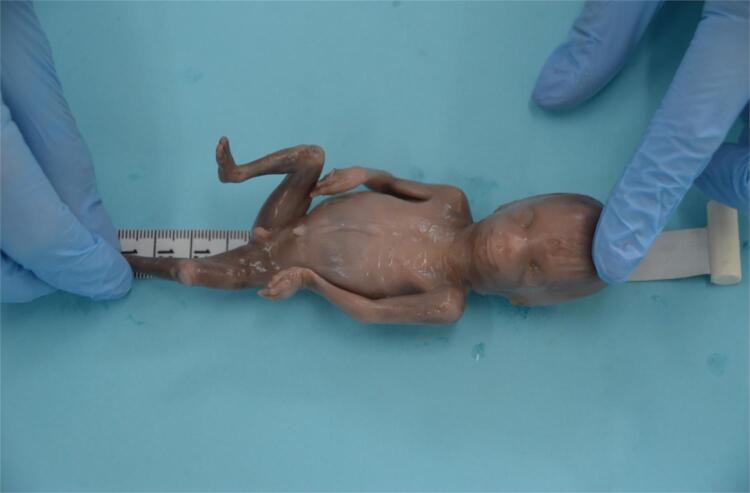
Fetal morphometric measures of the total length of a 22 weeks post conception fetus using a metric tape.

**Figure 2:**
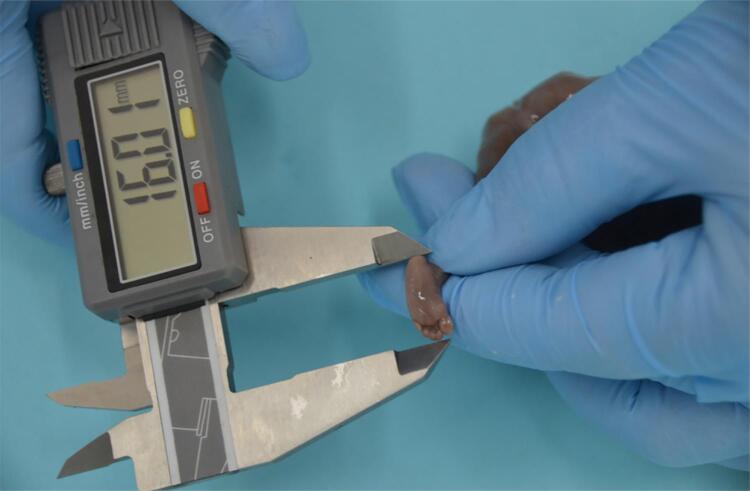
Precision pachymeter to check the measure of the bigger foot from the most prominent toe to heel. This measure is the most important for the determination of the gestational age.

The first step of fetal investigation applied to translational research in pediatric urology is the determination of fetal age. After determination of death, the fetuses are kept in refrigeration (temperature lower than 4 centigrade grades) for 24 to 72 hours. After reaching the laboratory, the fetuses are defrosted, cleaned, identified and analyzed morphologically. Fetuses with malformations or not well preserved are excluded for analysis. After cataloguing, the first step is to weight, using a precision scale of 1 gram. The fetuses are also evaluated regarding crown-rump length (CRL), total length (TL) and foot-length immediately before dissection ( Figure-1 ). The same observer analyses all measurements. For the evaluation of the CRL and TL it is used a metric tape, and to check the length of the bigger foot (more posterior region from the heel to the tip of the most prominent toe, first or second) it is used a Starrett® digital pachymeter 0.01 cm precision ( Figure-2 ). The measures of the right and left feet are repeated three times each, using the millimeter precision pachymeter (mm) ( 1 , 2 ).

The foot with the higher median is used to determine the gestational age, lowering the risk of error. That measure is analyzed in a graphic ( 3 ) that relates the length of the bigger foot with gestational age, according to weeks after conception (WPC). The gestational age of the fetuses is determined in WPC, according to the foot-length criterion, which is currently considered the most acceptable parameter to calculate gestational age ( 3 - 7 ).

**Figure 3:**
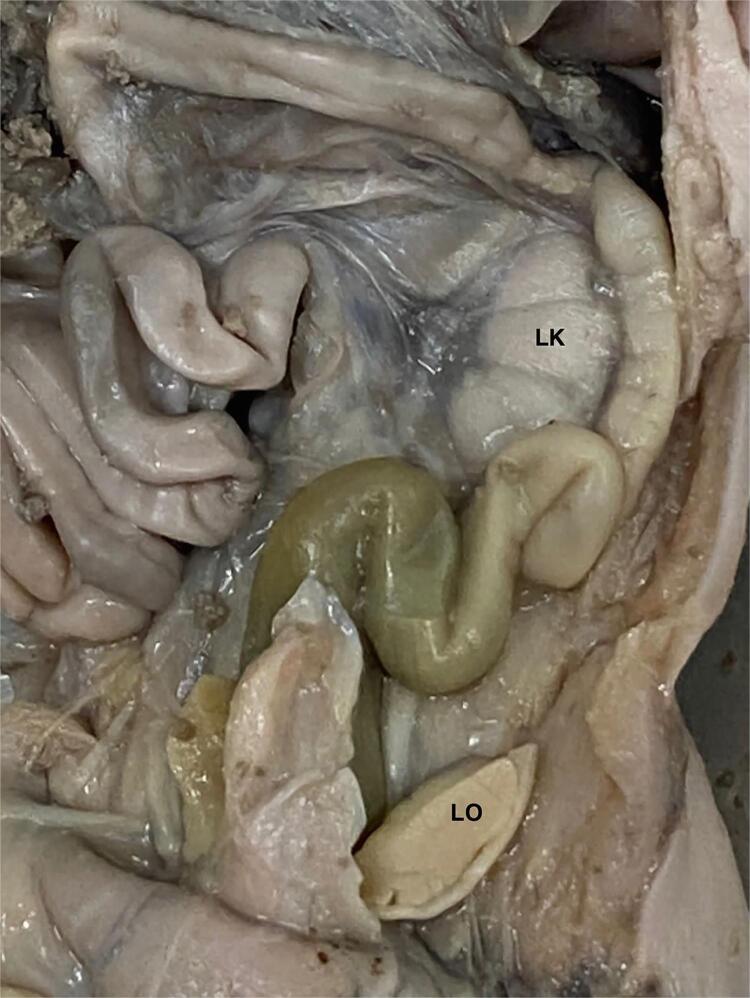
The figure shows the steps of fetal dissection of urogenital organs. The abdominal wall of a female fetus with 23 weeks post conception is opened and the urogenital organs (Left kidney –LT and Left ovaries –LT) are dissected for posterior histological analysis.

After the fetal measurements, the fetuses are carefully dissected with the aid of a stereoscopic lens with 16/25X magnification. The abdomen and pelvis are opened to identify and expose the urogenital organs ( Figure-3 ) and take the organs to histologic analysis. The samples are separated from the other structures and fixed in 10% buffered formalin, and routinely processed for paraffin embedding, after which 5-µm thick sections are obtained at 200-µm intervals. Smooth muscle and connective tissue, elastic system fibers and collagen are studied by histochemical and immunohistochemical methods. Sections are stained with hematoxylin-eosin to assess the integrity of the tissue. The following staining methods are used: Masson’s trichrome, to quantify connective and smooth muscle tissue; Weigert resorcin fucsin with previous oxidation, to observe elastic system fibers; and picrosirius red with polarization for observation of different collagen types. The main used immuno-histochemistry technique is avidin-biotin to identify collagenous proteins, elastin, and glycoproteins ( 8 ) and tubulin (Tubulin, beta III, Mouse Monoclonal Antibody) for nerves analysis.

**Figure 4:**
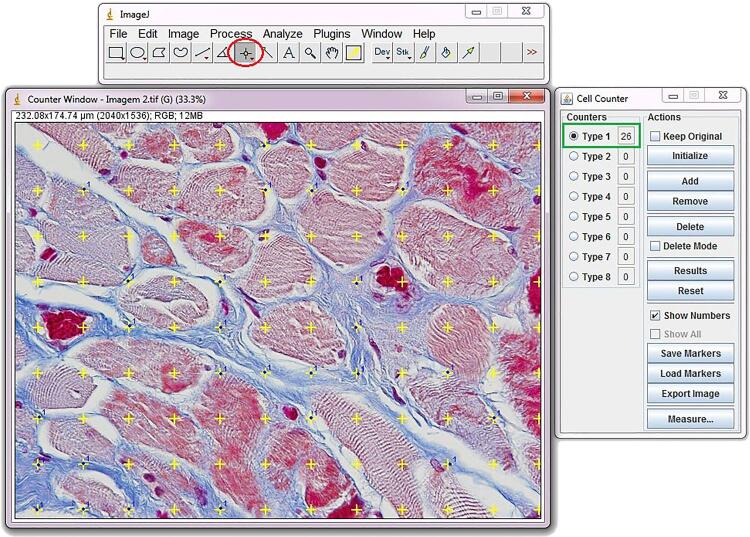
Quantification methods: To quantify the smooth muscle tissue we use the Color Segmentation of Image J software, where the program selects structures of different colors and calculates the amount of each component. After calibration and measure of the image area (red circle 39737.034), select the options plugins, analyze, and grid.

Connective tissue, smooth muscle tissue, nerves and elastic system fibers are quantified by a stereological method ( 9 ). We study 5 microscopic fields chosen at random, totaling 25 test areas studied for each gubernaculum for the quantitative analysis. We use the Image J software, version 1.46r, loaded with its own plug-in (http://rsb.info.nih.gov/ij/). All sections are photographed with a digital camera (DP70, Olympus America, Inc., Melville, New York) under the same conditions at a resolution of 2,040 1,536 pixels, directly coupled to the microscope (BX51, Olympus America, Inc.) and stored in a TIFF file. To quantify the smooth muscle tissue, we use the Color Segmentation of Image J software, where the program selects structures of different colors and calculates the amount of each component ( Figures-4 ).

**Figure 5:**
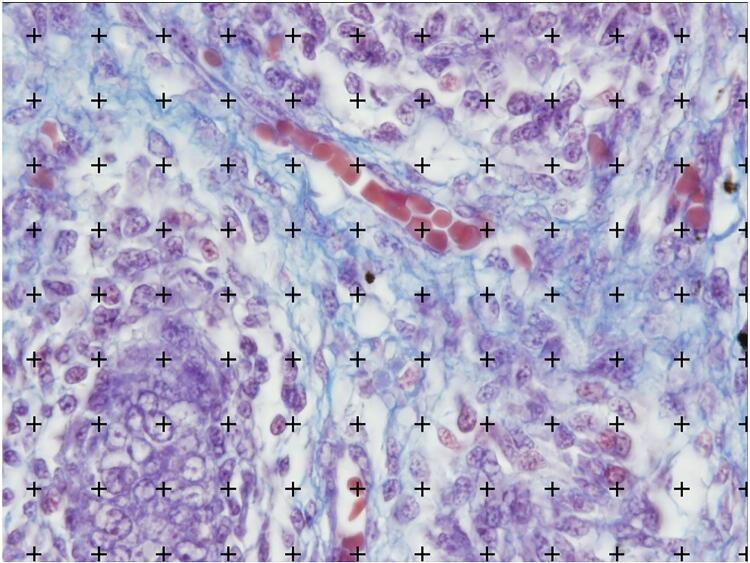
Quantification of muscle tissue with color segmentation of Image J software - grid window configuration overlapping the microphotograph.

For quantification of elastic fibers and nerves we use the Image J software to determine the volumetric density (Vv) of each component. Results for each field are obtained through the quantification assessment method, by superposing 100 points test grid (multipurpose test system) on the video monitor screen. The arithmetic mean of the quantification in 5 fields of each section is determined. Afterwards, we obtain the mean quantification value for the 5 sections studied from each sample (total of 25 test areas) ( Figure-5 ).

In order to quantify the area of collagen fibers, elastic fibers, blood vessels and nerves, it is used a plug-in cell counter and a point tool, that allows for the quantification of more than one structure in the same photography. The quantity of each analyzed structure is presented in the cell counter window, where the values are tabled, and the media obtained for each patient for statistical analysis.

For the analysis of the connective tissue and elastic system fibers it is used photography of the slices stained by the histochemistry techniques: Masson trichrome and Weigert resorcin-fucsin with previous oxidation, respectively. In both analyses, the microphotographs are obtained under 600X, and five random fields are analyzed by section. For the analysis of blood vessels and nerves it is used microphotographs of slices stained by the immune-histochemistry method: immune-labeling with anti-CD31 and anti-tubulin βIII, respectively. In both analyses, the microphotographs are obtained under 400X, and five random fields are analyzed by section, totalizing 35 fields in control group and 70 fields in the stained group.

**Figure 6:**
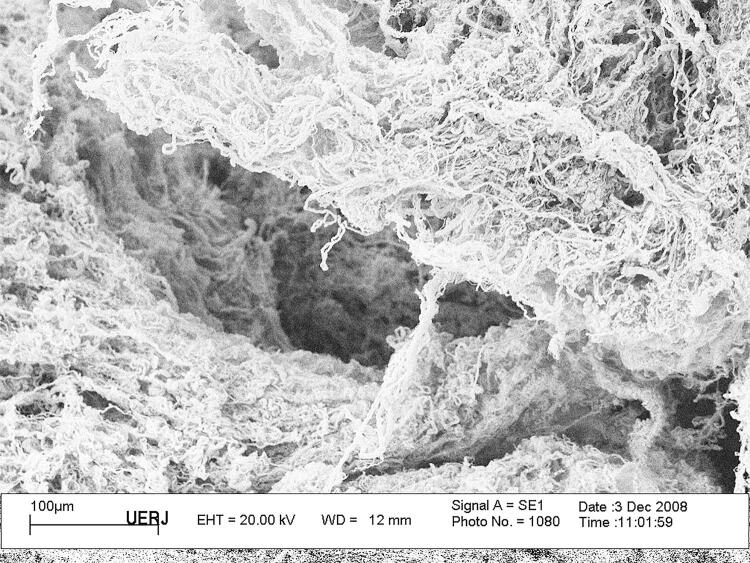
Scan electronic microscopy of fetal renal pelvis. Fetal renal pelvis of a male fetus with 18 weeks post conception.

For qualitative analysis of connective tissue, we studied 5 samples from each foreskin, with 2mm length. The samples are submitted to fixation for scanning electron microscopy (SEM) by immersing tissue fragments in a modified Karnovsky solution for 48 hours at 4ºC. This fixative consists of 2.5% glutaraldehyde and 2% paraformaldehyde in 0.1 M sodium phosphate buffer, pH 7.4. To better visualize the 3-dimensional organization of the vesicle stroma under SEM, tissue samples are submitted to an alkali treatment to solubilize and remove cells. The obtained acellular preparations are then processed for high-vacuum SEM, and observations are performed on a LEO 435 (Zeiss, Oberkochen, Germany) scanning electron microscope with an acceleration voltage of 15 to 20 kV ( Figure-6 ).

The injection corrosion techniques using resins and anatomic models are very important for translational research. These techniques allows for the tridimensional study of several organs, the study of micro-vascularization, analysis of anatomic relations in humans, and experiment and animal models ( 10 ). Resins are polymers capable of produce solid and saturated compounds (anatomic models). The ideal resin should be cheap, with minimal retraction, producing a strong and consistent mold with unchanged color and easy to manipulate. There are several kinds of resins: plastic material, synthetic resins and silicon resins. The plastic include nylon, vinilyte and Justi h. This kind of resin shows too much retraction (distortion), is fragile, changes its color and needs a high pressure for injection, complicating its routine use.

The synthetic resins include Resapol T208 and Perpex tensol, routinely used in our laboratory with great experience. This class of resin is very resistant to caustic agents. We obtained an easy viscosity regulation with minimal retraction, and they have a low cost. We routinely use Resapol. It is composed by resin, a styrene monomere, a catalyzing agent and a dye (pigment paste).

The styrene monometer allows for the co-polymerization and produces a mixture with good viscosity. The catalyzing agent (Ethyl-methyl-ketone peroxide) stiffens the resin, a fundamental step for the confection of molds. The catalyzing agent is liquid, easy to mixture, unstable, with a short limit time for use, and bubbles indicate deterioration.

**Figure 7:**
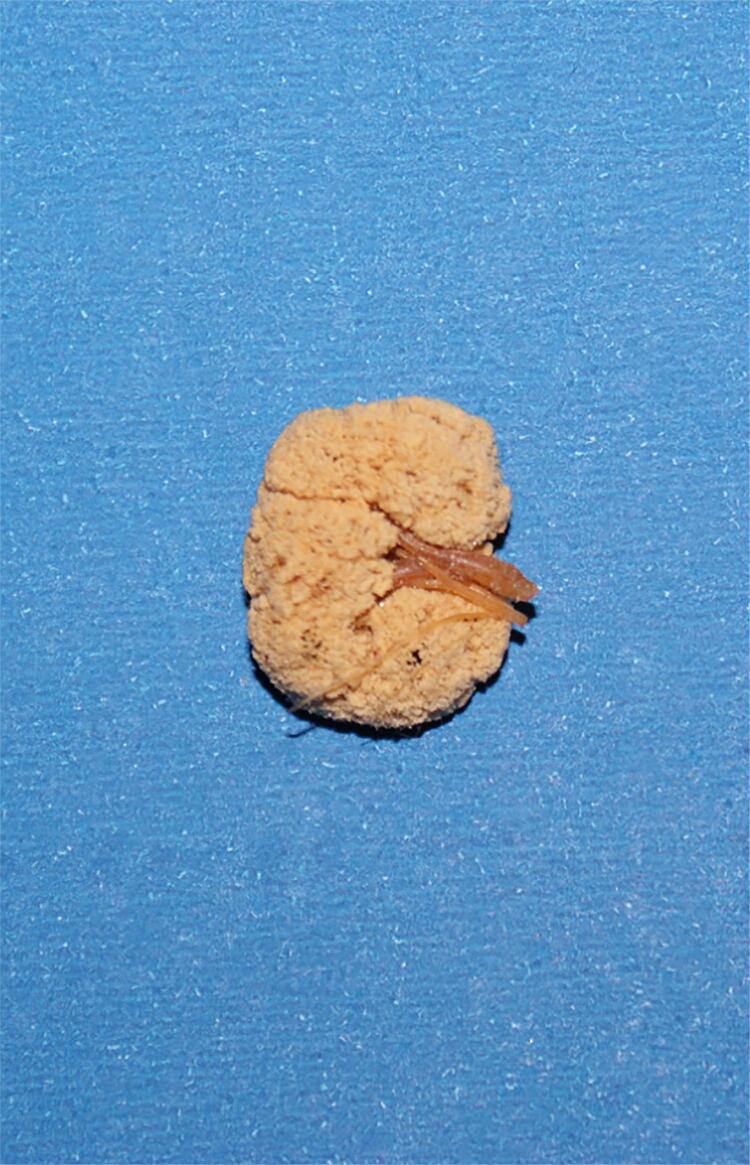
The figure shows the final aspect of a kidney endocast in a fetus with 25 weeks post conception.

In order to perform the injection, we use the following method: for each 100 ml of resin, we add 10 ml of styrene monomer and 2 to 5 ml of catalyzing agent, and the dye (we standardized the following colors: yellow for the collecting system, red for arteries and blue for veins). Following the resin hardening, we initiate the process of corrosion in order to remove all organic material and confection of the mold ( Figure-7 ). After injection, the material must be dipped in hydrochloric, sulfuric, or muriatic acids for 24 hours. After this time, the mold must be removed from the recipient, cleaned, and dried for analysis ( 10 ).

**Figure 8:**
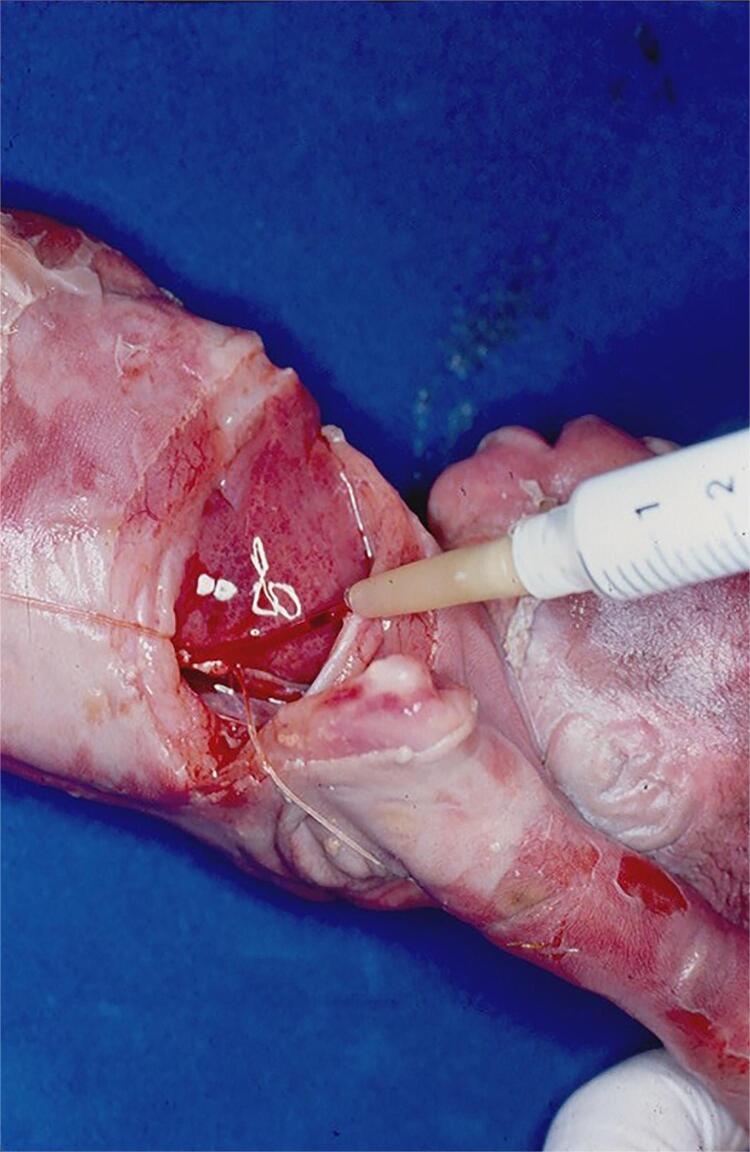
Silicone (Microfil) Resin injection Technique: The fetal thoracic cavity is open and the descendent aorta is catheterized and injected.

Silicon resin Microfil can also be used, particularly when the purpose is to highlight the organ vasculature. This kind of resin has high cost and is difficult to obtain. We use it in special to study the renal and testicular vasculatures. By thoracostomy, we identify the thoracic aorta and inject the resin inside the vessel ( Figures 8 ). After injection, the abdominal cavity is open and with the aid of a stereoscopic magnifying glass we carefully dissect the organ vessels.

The use of these techniques allows the development of several lines of research on the urogenital system during the human fetal period, which is of fundamental importance for translational medicine.
